# Verrucous carcinoma arising from a previous cystic lesion: a case report

**DOI:** 10.1186/s40902-018-0169-x

**Published:** 2018-10-25

**Authors:** Sunghyun Kang, Dae Ho Leem

**Affiliations:** 1 Department of Oral and Maxillofacial Surgery, Chonbuk National University Hospital, 20 Geonjiro, Deokjin-gu, Jeonju-si, Jeollabuk-do 54907 South Korea; 2 Department of Oral and Maxillofacial Surgery, School of Dentistry, Chonbuk National University, 567 Baekje-daero, Deokjin-gu, Jeonju-si, Jeollabuk-do 54896 South Korea

**Keywords:** Verrucous carcinoma, Intraosseous verrucous carcinoma, Primary intraosseous carcinoma, Odontogenic cyst, Maxilla

## Abstract

**Background:**

Verrucous carcinoma (VC) accounts for 1–10% of cases of squamous cell carcinoma (SCC) in the oral cavity, and 75% of VC occur in the oral cavity. Only 3% of primary intraosseous squamous cell carcinomas (PIOSCC), which means SCC occurring primarily in the bone, are VC. Verrucous carcinoma arising from odontogenic cysts (OC) is very rare, with only seven cases reported to date.

**Case presentation:**

This study reported a case of a patient who underwent partial maxillectomy and neck dissection for VC that occurred in the right anterior maxilla. The patient was admitted to the emergency department at our institution 8 years ago and showed cystic lesions in the anterior maxilla on facial computed tomography (CT) images. Treatment through other departments including assessment of laceration in the mental region and only suture was performed. This report highlights a very rare case of VC in the right anterior maxilla arising from a previous cystic lesion.

**Conclusions:**

Since PIOSCC can arise from OC, appropriate treatment of intraosseous cysts and regular radiologic evaluation are necesssary. Surgical exicision of the primary lesion without neck dissection can lead to good prognosis for patients with primary intraosseous verrucous carcinoma.

## Background

Verrucous carcinoma (VC), first reported by Ackerman in 1948, is characterized by exophytic and papillary friable lesions. It is slowly growing, well differentiated, and verrucous in nature and often extensive with a tendency to invade local structures. The main causes of VC are poor oral hygiene, smoking, and alcohol abuse [1, 2].

Kraus and Perezmesa reported that VC can occur in various epithelial lining, but is most common in the oral cavity, larynx, esophagus, and genitalia [3]. Verrucous carcinoma is characterized by diffuse and exophytic lesions covered by leukoplakic patches. It grows slowly over several years and forms a cauliflower-like lesion 3.0–5.0 cm in size with indolent pain within a few months after turning into a small wart-like mass. It shows aggressive local invasion of associated structures such as the bone and cartilage around the primary lesion and rarely spreads to the lymph nodes. Verrucous carcinoma has relatively good prognosis due to its low metastatic and invasive potential and is treated by surgical excision without radical neck dissection [4].

Carcinoma within the bone is a very rare form of VC and often arises from odontogenic cysts (OC) [4]. Borrás-Ferreres et al. reported the rate of carcinomatous transformation of OC of 0.13–3% [5]. Carcinomatous transformation of OC is characterized by pain and swelling, but may be detected on panoramic radiographs without accompanied symptoms. Histologically, OC transformation to well-differentiated SCC is reported as the most common finding. With regard to OC transformation to SCC, Schwimmer et al. reported that 60% of SCC arose from residual or radicular cysts, 35% arose from dentigerous cysts or odontogenic keratocytes, and 11% arose from lateral periodontal cysts and other types of cysts [6]. Bodner et al. reported 116 cases of primary intraosseous squamous cell carcinoma (PIOSCC) which means SCC occurring primarily in the bone that occurred at OC [7]. Primary intraosseous squamous cell carcinoma refers to carcinoma originated in the jaw as primary site of lesion and accounts for 1–2% of all OC. Roughly 85% of PIOSCC is well-to-moderately differentiated SCC, and only 3% is VC. VC arising from OC is a type of PIOSCC and is referred to as primary intraosseous verrucous carcinoma (PIOVC) [8].

Verrucous carcinoma arising from an OC is very rare, with only seven cases reported to date. Diagnosis of carcinoma originating in the jaw, especially carcinoma arising from an OC, is difficult because it is necessary to determine if the carcinoma is primary, originating from another site or spreading through the alveolar bone if the carcinoma occurs on the mucous membrane, or originating from the maxillary sinus. In case of carcinoma arising from an OC, it is difficult to confirm through histology that the carcinoma originated from OC. Therefore, there are very few cases of carcinoma arising from OC reported to date, and VC arising from OC is extremely rare [9].

This study reported a case of partial maxillectomy and neck dissection performed for VC that occurred on the labial surface of the edentulous alveolar ridge in the anterior maxilla and in the middle of the palate. Facial CT imaging 8 years ago indicated that the patient had an odontogenic cystic lesion on the ipsilateral side. At the time, the patient visited the emergency department at the present institution for laceration in the mental region and underwent suturing. The patient underwent assessment for the presence of jaw fracture subsequent to the facial CT but was not informed regarding the prognosis of or treatment for the cystic lesion in the maxilla. This case report highlighted the possibility of VC arising from a previous cystic lesion, with a comparative review of similar cases.

## Case presentation

A 76-year-old patient visited a private clinic in May 2016 after developing a lesion inside his mouth. Incisional biopsy showed a lesion on the labial surface of the edentulous alveolar ridge in the anterior maxilla, and the patient was diagnosed with VC. The patient was referred to the Department of Oral and Maxillofacial Surgery at Chonbuk National University Dental Hospital in June 2016.

**Fig. 1:**
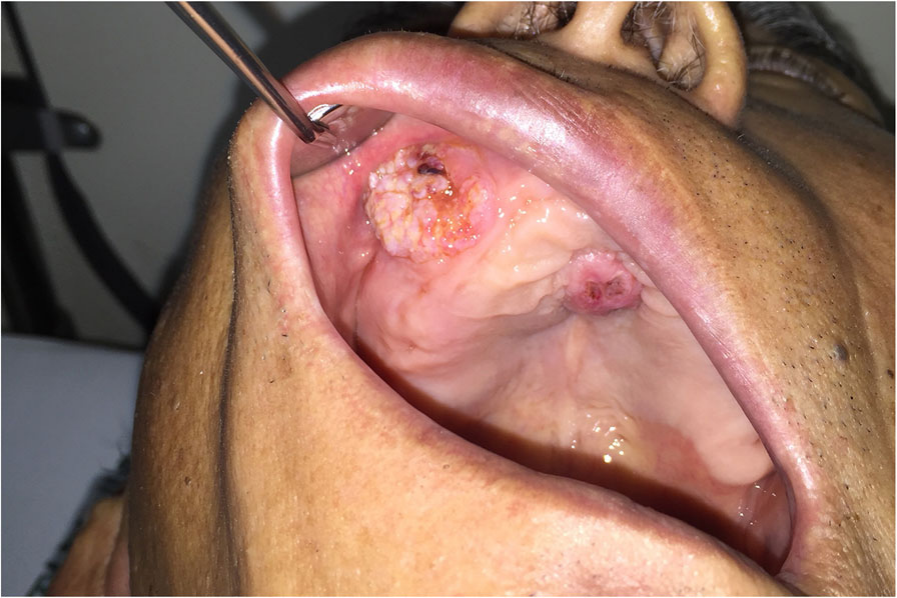
Clinical photograph showing papillary exophytic growths of the labial parts of the maxillary right anterior edentulous ridge and middle of the palate

**Fig. 2:**
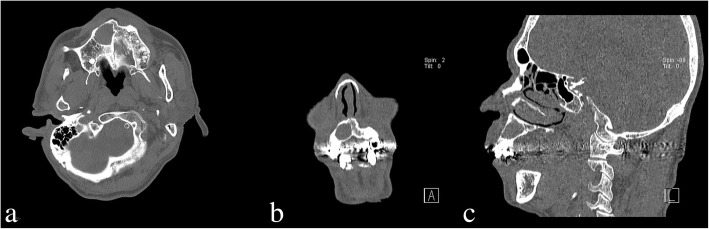
Facial CT image acquired 8 years ago at the emergency room showing a well-circumscribed, corticated, expansile, unilocular, and radiolucency lesion in the right anterior maxillary area. **a** Axial view. **b** Coronal view. **c** Sagittal view

At the time of admission, the patient showed painless exophytic verrucous proliferation measuring 2 cm × 2 cm on the labial surface of the edentulous alveolar ridge in the anterior maxilla and 1 cm × 1 cm in the middle of the palate. The lesions’ surfaces were covered with thick, white keratin (Fig. 1). The patient had been diagnosed with hypertension and prostatism 6 years ago, which were being controlled through medication. The patient had stopped taking aspirins 3 months ago. At the time of visit to the Emergency Department of Chonbuk National University Dental Hospital for laceration in the mental region 8 years ago, facial CT through referral from another department indicated the presence of cystic lesion surrounded by the unperforated cortical bone in the right anterior maxilla (Fig. 2). However, through facial CT performed at the time of the admission to the Emergency Department explained to the patient only about there was no facial bone fracture. The patient reported that he had not been informed of the presence of cystic lesion in the maxilla at the time of visit to the emergency department 8 years ago and not received any treatment due to absence of symptoms. The patient underwent full-mouth extraction for the remaining maxillary and mandibular teeth 3 months before visiting the private clinic and has been wearing maxillary and mandibular complete dentures since then.

**Fig. 3:**
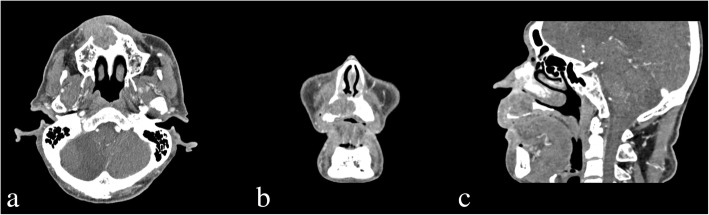
Facial CT image acquired at the first visit in 2016 showing a dome-shaped radiolucent area in the right maxilla with expansion to the nasal floor and jagged margin of the cortical bone. **a** Axial view. **b** Coronal view. **c** Sagittal view

**Fig. 4:**
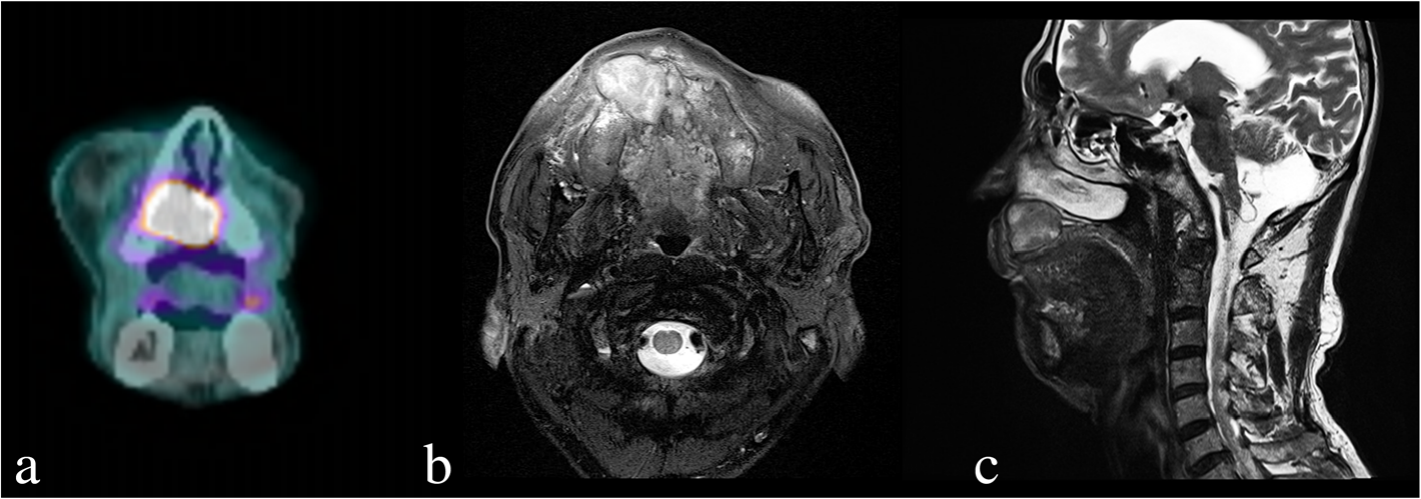
PET and MRI scans acquired before surgery. **a** 18-FDG PET/CT coronal view showing hot FDG spot on the right maxillary area. **b** T2-weighted MRI scan, axial view. **c** T2-weighted MRI scan, sagittal view

Based on results from incisional biopsy of the lesion performed at Chonbuk National University Dental Hospital at the time of admission, the patient was diagnosed with VC. Since the time of initial detection 8 years ago, the cystic lesion in the right maxilla was more widespread, with surrounding bone destruction, unclear border, and irregularly absorbed nasal mucous, anterior maxilla and palatine bones (Fig. 3). MRI test showed heterogeneous enhancement in the right maxilla with accompanied bony erosion, with invasion of the levator labii superioris muscle, and zygomatic muscle in the anterior direction (Fig. 4). Heterogeneous enhancement with maximum 1 cm radius was observed in the lymph node at the right cervical chain levels I and II, so metastasis was suspected. On FDG PET/CT, an enlarged lymph node with FDG uptake was observed at the right cervical levels IB and II. Supraomohyoid neck dissection (SOHND) was planned under suspicion of invasion of the neck.

Findings from chest radiographs, electrocardiograms, and blood tests presented no contraindication for general anesthesia, in addition, the chest radiographs revealed absence of specific lesions.

**Fig. 5:**
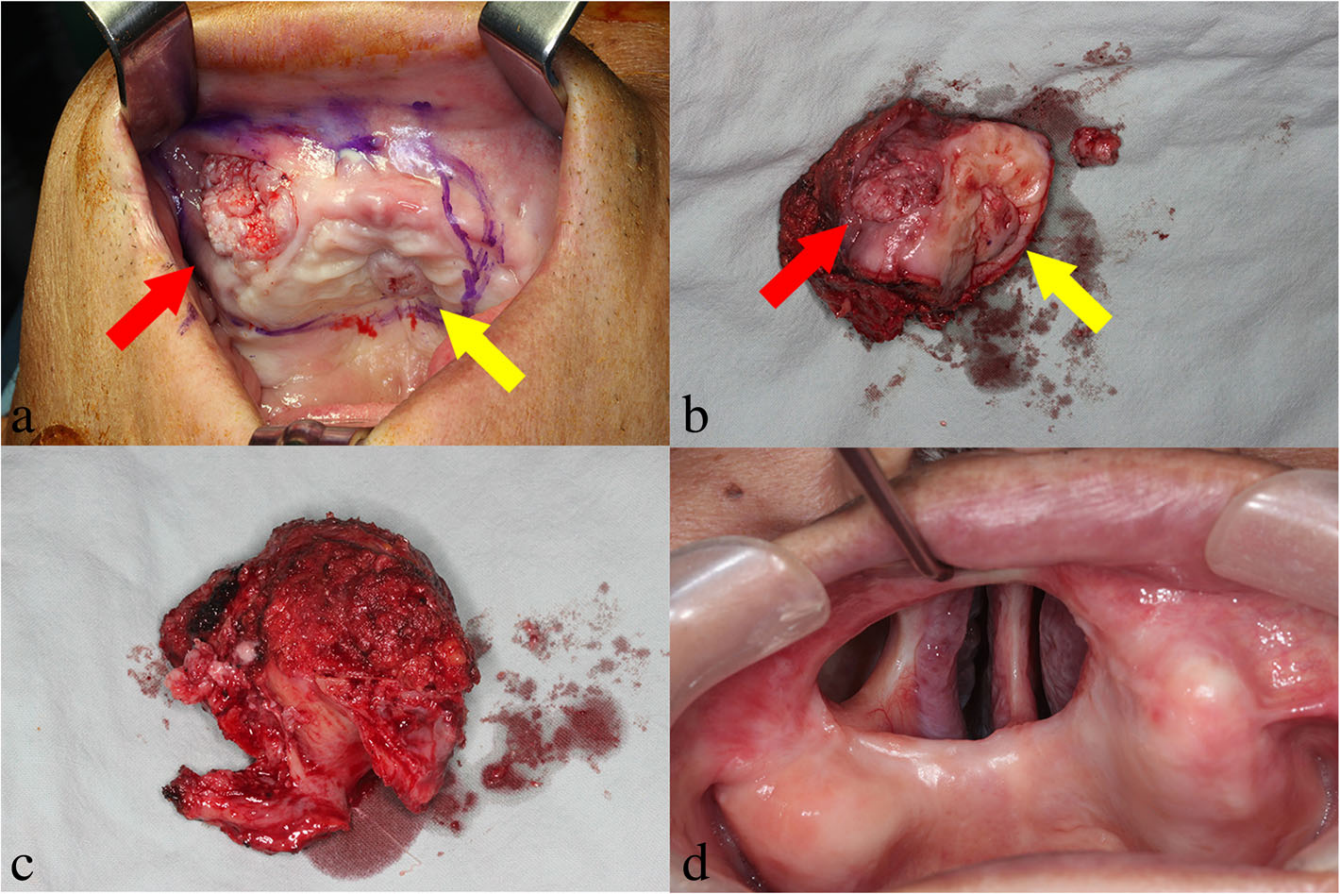
Preoperative and postoperative clinical photograph. **a** Pre-operative image. **b** Excisional specimen (the right side of the maxilla, red arrow; the palatal side, yellow arrow). **c** Verrucous proliferation pattern can be seen on the surface of the lesion inside the bone. **d** At 2-years post-surgery, there is no recurrence of findings

In July 2016, partial maxillectomy of the right maxilla and SOHND were performed (Fig. 5) under general anesthesia. Partial maxillectomy with safety margin of 1 cm and frozen biopsy were performed; results showed negative findings. In addition, result from frozen biopsy of the enlarged lymph node showed negative findings. Furacid gauze packing was performed at the site of partial maxillectomy, and a premade obturator was fixed on the maxilla through using a screw.

Cross-sectional image of the excised right maxillary lesion showed presence of a solid mass with unclear border surrounding the tissues and with white papillary appearance. In addition, the lesion on the edentulous alveolar ridge of the right anterior maxilla and the lesion in the middle of the palate was found to be a single exposed mass within the oral cavity. Finally, the patient was diagnosed with VC based on the biopsy results. The lesion was staged as pT4aN0 since no metastasis was observed in the lymph nodes.

**Fig. 6:**
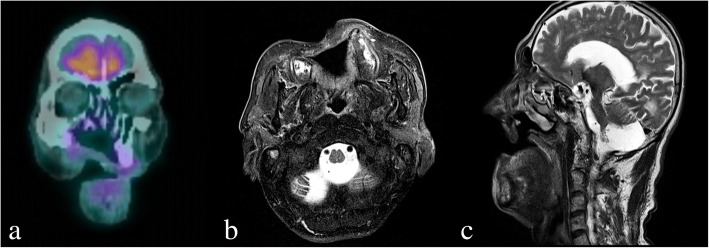
PET and MRI scans acquired 6 months after surgery indicate no recurrence of findings. **a** 18-FDG PET/CT coronal view showing only slight spots around the partial maxillectomy area due to the surgery. **b** T2-weighted MRI scan, axial view. **c** T2-weighted MRI scan, sagittal view

**Fig. 7:**
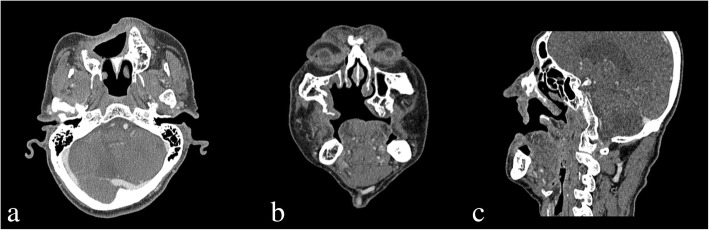
Facial CT image acquired 1 year after surgery indicates no recurrence of findings. **a** Axial view. **b** Coronal view. **c** Sagittal view

Follow-up evaluation at 6 months (Fig. 6), 1 year (Fig. 7), and 2 years (Fig. 5d) after surgery revealed the absence of recurrence or metastasis.

### Histopathologic findings

**Fig. 8:**
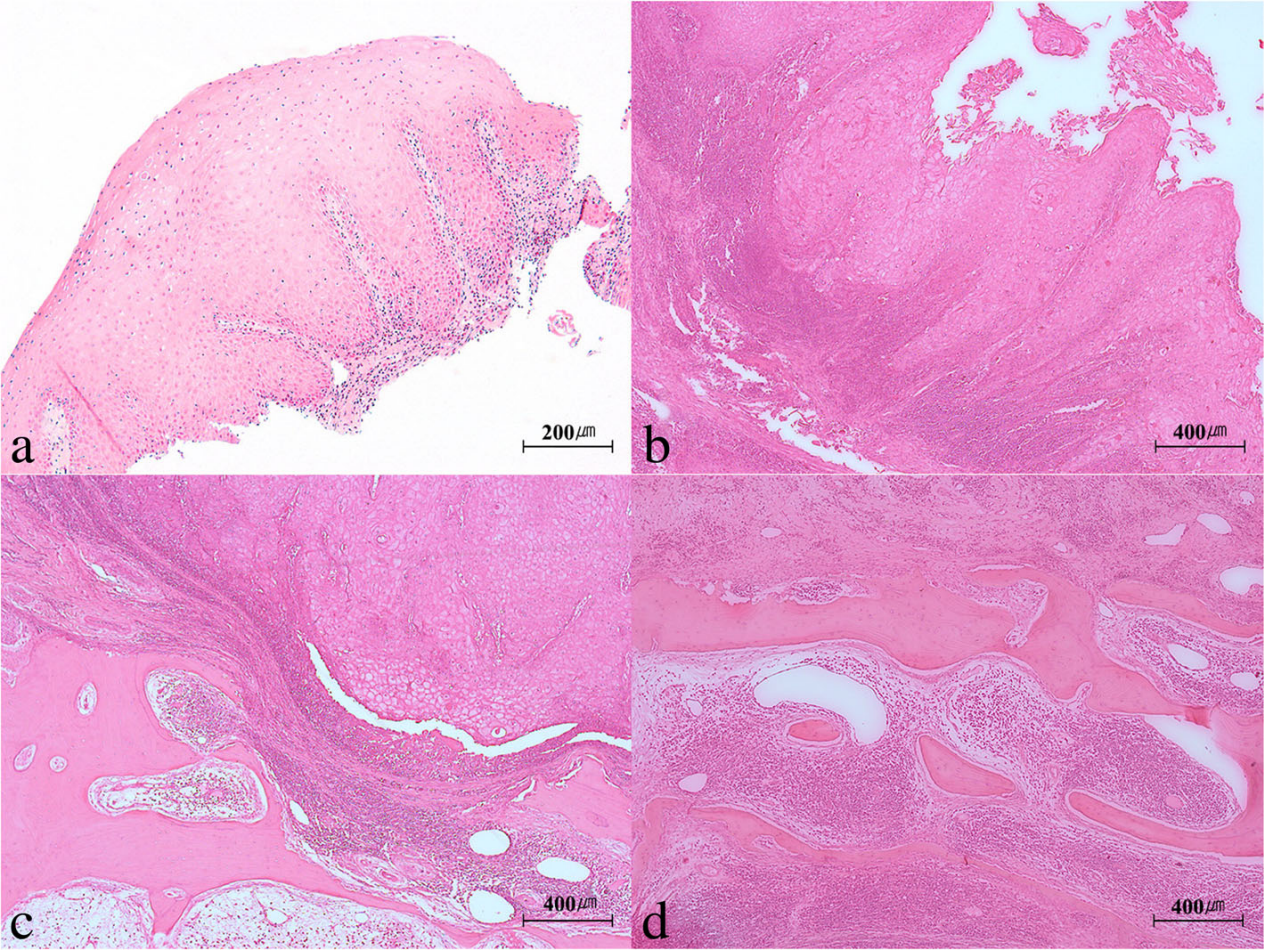
Histopathologic microphotographs of the specimen with hematoxylin and eosin staining. **a** Incisional biopsy before surgery showing downward growth of numerous thickened, bulbous rete ridges of squamous cells (× 100). **b** Surgical specimen showing exophytic growth pattern with prominent keratin layer arranged in compressed invaginating fold (× 50). **c** Hyperplastic epithelium showing blunt projections rather than infiltration of the underlying tissue with dysplasia and hyperplasia of the basal cell layer (× 50). **d** A group of epithelial cells proliferating within the maxillary bone with bony destruction (× 50)

An incisional biopsy of the lesion in the right maxillary alveolar ridge performed at the present institution showed downward growth of numerous thickened and bulbous rete ridges of squamous cells. With regard to biomarker testing, the lesion was p16 positive and had lower proliferation based on Ki-67 detection; as a result, the patient was initially diagnosed with VC (Fig. 8a). Histological examination performed after surgery showed epithelial cells with exophytic surfaces and prominent keratin layer arranged in compressed invaginating folds that proliferated downward (Fig. 8b). In addition, blunt club-shaped projections of hyperplastic epithelium rather than infiltration were observed, and the basal layer exhibited dysplasia and hyperplasia (Fig. 8c). Groups of epithelial cells proliferating within the maxilla associated with bony destruction was observed (Fig. 8d). Finally, the patient was diagnosed with VC based on the observation of exo-endophytic growth pattern, deep extension of thick, parakeratotic, squamous epithelial cell layer, and bulbous rete ridges with borders extending toward the adjacent tissue with locally invasive patterns.

The diagnosis of VC is based on histopathological findings as well as clinical symptoms, since the biopsy specimen does not always represent the full thickness of the epithelium [10]. Our patient exhibited clinical and histopathological characteristics of VC including epithelial cells with associated destruction of the bone within the maxilla and proliferation with combined dysplasia and hyperplasia. Since the patient had a cystic lesion in the same area, it was concluded that the patient had VC arising from a previous cystic lesion.

## Conclusions

The remaining epithelium after odontogenesis proliferates and differentiates due to unknown stimuli and forms an odontogenic tumor. The cysts or tumors may undergo metaplastic transformation into squamous epithelium to develop into malignant tumors [11].

Gardner analyzed 63 cases of carcinoma arising from OC reported since the first report of carcinoma arising from OC by Herrmann until 1967 and reported that long-standing chronic inflammation is the major factor promoting the transformation of cysts to carcinoma [12].

In 1971, the WHO defined primary intraosseous carcinoma as SCC within the jaw that originates from remnants of odontogenic epithelium rather than the oral mucosa [13]. Although there is controversy regarding the cause of carcinomatous transformation of OC, most studies explain that long-standing chronic inflammation may act as a malignant transformation factor, and that the connective tissue infiltration of lymphocytes and plasma cells may be the major cause of transformation.

In 1963, Ward and Cohen classified the causes of carcinoma arising from OC: first, metastasis of carcinoma from another site to an existing cyst; second, transformation of carcinoma into cyst; and third, malignant changes of the cyst [14].

Gardner proposed the following criteria for SCC originating from OC in 1975: microscopy findings of transition area from benign cystic epithelial lining to invasive SCC, absence of carcinomatous changes in the overlying epithelium, and no source of carcinoma in the adjacent structures [12]. To et al. proposed the following criteria for PIOSSC diagnosis in 1991: histology finding of intrabony SCC with no evidence of associated oral disease, negative finding on chest radiograph, and 6 months’ survival with no evidence of occult primary or negative autopsy [15]. Suei et al. explained that the possibility of occurrence of primary tumor in another site must be ruled out in order to diagnose PIOSCC [9]. However, since the causal relationship is unclear, a follow-up period of at least 6 months that includes chest radiography is necessary to rule out the possibility of lesions from another site [15].

Mucosal changes or metastasis from another lesion should be ruled out for the diagnosis of PIOSCC arising from OC. Differentiating between the origins of histologically similar PIOSCC at OC and PIOSCC that metastasized from a distant lesion such as the mucosal membrane, or that of highly advanced PIOSCC is difficult [16]. Therefore, a radiologic examination is useful for diagnosing PIOSCC. A lesion with radiographic findings of surrounding bone can be assumed to be of intraosseous origin [9].

Whereas, OC generally presents as a round or oval radiolucent lesion with well-defined borders on radiographs; OC that has undergone malignant transformation shows jagged margins, indentations, and indistinct borders. In addition, it is characterized by bony expansion with thinning of the cortex of the maxilla and mandible [12]. It also shows resorption of the adjacent dental roots in addition to bone erosion in the buccal (labial) and palatal (lingual) sides [17].

Bodner et al. reviewed 116 cases of PIOSCC [7]. However, VC arising from OC is very rare, with only seven cases reported to date (Table 1) [2, 4, 8, 18–21].

**Table 1:** Reported cases of verrucous carcinoma arising from odontogenic cystic lesion

Author (year)	Age/sex	Site	Symptom	Diagnosis	Prognosis, follower-up period, month
Enriquez et al. (1980) [2]	56/M	Mandible	Painless lesion, fistulous tract	VC arising from OC	No recurrence or metastasis, 48
Anand et al. (1994) [18]	46/M	Maxilla	Discomfort	VC arising from OKC	No recurrence or metastasis, 24
Pomatto et al. (2001) [8]	NA/F	Maxilla	Recurrent swelling	VC arising from DC	No recurrence or metastasis, 8
Mohtasham et al. (2008) [4]	58/M	Maxilla	Non-tender on palpation, exophytic lesion	VC arising from OKC	No recurrence or metastasis, 20
Imaue et al. (2013) [19]	52/M	Maxilla	Painless exophytic lesion	VC arising from POC	No recurrence or metastasis, NA
Peng et al. (2015) [24]	74/M	Mandible	Swelling with mild pain	VC arising from DC	No recurrence or metastasis, 5
Kamarthi et al. (2016) [21]	65/M	Maxilla	Painful swelling	VC arising from OKC	No recurrence or metastasis, 6
Present case (2018)	76/M	Maxilla	Painless exophytic lesion	VC arising from POC	No recurrence or metastasis, 24

*VC* Verrucous carcinoma, *OC* odontogenic cyst, *OKC* odontogenic keratocyst, *DC* dentigerous cyst, *POC* previous odontogenic cystic lesion, *NA* not available

Takeda reported that the risk of malignant change of OC was four times higher in the mandible than in the maxilla [22]. In addition, the risk of VC is reported to be two times higher in the mandible than in the maxilla and four times higher among men than women, with the mean age at onset of 53.9 years [23]. However, of the total eight reported cases of OC transforming to VC including the present case, six cases showed transformation in the maxilla (75%), and seven cases were male (87.5%). The mean age at onset of VC was 61.0 years.

### Characteristics of each reported case

Enriquez et al. reported the first case in 1980 of VC at OC in the mandible [2]. The second case was by Anand et al. in 1994 on VC in the maxilla with odontogenic keratocysts [18]. The third case was reported by Pomatto et al. in 2011, on VC at OC in the maxilla following tooth extraction in the left posterior maxilla; the authors claimed that VC originated from post-extraction remnants and introduced the term PIOVC [8]. The fourth case was reported by Mohtashm et al. in 2008, on VC at OC in the maxilla [4]. Mohtashm et al. explained that since metastasis of VC is extremely rare, surgical exicision without radical neck dissection is the best treatment option for VC. The fifth case was reported by Imaue et al. in 2013, on VC at OC in the maxilla [19]. Similar to the present case, a dome-shaped radiopaque mass with well-defined margins was observed in the left maxilla on radiograph, with expansion toward the maxillary sinus, nasal septum, and zygomatic arch. Partial maxillectomy was performed. The sixth case was reported by Peng et al. in 2015, on VC at OC in the mandible [24]. Peng et al. summarized all reports regarding VC and explained that since PIOVC has no recurrence or metastasis, it does not require any forms of neck dissection, and removal of primary lesion alone can lead to good prognosis. The seventh case was reported by Kamarthi et al. in 2016, on VC at OC in the maxilla with confirmed verrucous transformation of the cystic lining [21].

Early histological findings of VC include keratin piling on the surface, with initiation of downgrowth of epithelial projections. With progression of VC, club-shaped fingers of hyperplastic epithelium that gradually project into rather than infiltrate the deeper tissues are observed. At this point, the epithelium is well-differentiated while the basement membrane is intact. With growth of VC, cleftlike spaces with degenerating keratin project deeply. Chronic inflammation of the connective tissues, plasma cells, and mononuclear cells may ensue [1].

Odontogenic cyst that has undergone malignant transformation is characterized by chronic infiltration of lymphocytes and plasma cells in the connective tissue of the cyst wall. Cyst walls exhibit hyperplastic epithelium, and tumor cells exhibit pleomorphism and hyperchromatism. Cholesterol crystal clefts may be observed, and they may invade fibrous cystic walls. The cyst walls may have normal epithelial surface on some parts and naive epithelial surface on the others [12].

Maxymiw and Wood reported a case of OC advanced to SCC in the mandible, and based on the finding of transition of the cellular lining of the cyst wall from benign epithelium to carcinoma, it was confirmed that OC originated from carcinoma. In this case, malignant transformation occurred through chronic infiltration by lymphocytes and plasma cells of the cyst walls’ connective tissues. Cysts are partially or fully lined with dyskeratotic squamous epithelium. The stratified squamous epithelium with linings can transform to invasive SCC from the normal squamous epithelium and proliferate into the connective tissues of the cyst [17].

Verrucous carcinoma arising from OC is characterized by the transformation of the lining epithelium of the cyst to verrucous hyperplastic epithelium. In addition, moderate chronic inflammatory cells infiltrate the fibrous cystic wall. Proliferation of hyperparakeratotic-stratified squamous cyst lining epithelium can occur, and down-growth of broad and bulbous epithelial ridges with pushing-border invasion, which is generally observed in VC, can occur in the fibrous cystic wall. Thick prominent rete pegs with well-preserved basement membrane and well-differentiated squamous cells which penetrate deeply into the underlying tissue are also observed. Cell nuclei is prominent, and cellular pleomorphism is observed, with mild dysplasia, focal dyskeratosis, and atypical squamous cells. In addition, mitotic figures of the basal and parabasal epithelial cells are observed [2, 20].

Most reports indicate that surgical excision of the primary lesion is sufficient for VC, and VC shows higher 5-year survival rate than SCC. McClure et al. reported that although more aggressive excisions are necessary for large tumors, neck dissection is not necessary, since in most cases, VC is inflammatory rather than metastatic even in cases with lymphadenopathy at preoperative examinations [10]. However, Walvekar et al. recommended that considering the uncertainty of pathological diagnosis in cases with suspicious lymphadenopathy, it is reasonable to perform selective neck dissection such as SOHND. According to his analysis of 101 cases of VC within the oral cavity, the rate of recurrent VC was 28%, and the 5-year survival rate after surgery was 77.6% [23].

Ackerman reported that while radiotherapy may be effective for small superficial VC, surgical excision must be accompanied for cases with VC affecting a large area [1]. Few studies have reported that irradiation of VC, which is radiosensitive, can cause high-recurrence rates and anaplastic transformation of the neoplasm leading to rapid metastatic dissemination [3, 10].

Gardner analyzed 25 cases of carcinoma arising from OC and reported patients’ mortality rate of 20% within 10–24 months after surgery [12]. In studies including 27 cases each of carcinoma arising from OC, Eversole et al. reported a 2-year survival rate of 53% and Schiwmmer et al. that of 63% [6, 25]. In a study including 116 cases, Bodner et al. reported that 46% of cases with PIOSCC underwent surgery, and 38% underwent surgery and radiotherapy; follow-up revealed a 2-year survival rate of 62% and 5-year survival rate of 38%; and only 6 out of 116 cases with neck metastasis; however, despite the low rate of neck metastasis, neck dissection was performed in 59 of 116 cases (51%) [7].

In the seven reported cases of VC arising from odontogenic cystic lesion, excluding the present case report, neck dissection and radiotherapy were not performed. In this case report, neck dissection was performed due to PET/MRI characteristic of enlarged lymph node, but radiotherapy was not performed. Surgical excision of the primary lesion led to good prognosis. As suggested by the criteria, VC which is less malignant than SCC, with primary lesion in the jaw without distant lesion, low metastatic potential, and low recurrence rate may lead to good prognosis.

Our patient showed cystic lesion surrounded by the cortical bone at the site of carcinoma since 8 years ago, clear histological characteristics of VC, bony destruction of the maxilla, and cancer cell infiltration of the jaw; based on these findings, the patient was diagnosed with PIOVC. However, based on the diagnostic criteria for PIOSCC, the presence of radiolucent cystic lesions at the site of VC observed 8 years ago, characteristics of the carcinoma cells differentiating within the bone, and absence of distant lesion, as well as previous reports, the patient was diagnosed with VC arising from odontogenic cystic lesion.

In conclusion, this case report highlights that patients with OC must be promptly diagnosed and managed due to the possibility of malignant transformation of OC. Careful monitoring of lesions through CT is necessary for patients with OC. In addition, their prognosis must be carefully observed through regular follow-up x-ray examination. As reported in previous studies, surgical excision of the primary lesion without neck dissection can lead to good prognosis of PIOVC. However, if a patient exhibits combined lymphadenopathy, the possibility of future occurrence of metastasis must be completely eliminated through selective neck dissection.

## Abbreviations

18-FDG PET/CT: Positron emission tomography with 2-deoxy-2-fluorine-18-fluoro- D-glucose integrated with computed tomography
CT: Computed tomography
MRI: Magnetic resonance imaging
OC: Odontogenic cyst
PIOSCC: Primary intraosseous squamous cell carcinoma
PIOVC: Primary intraosseous verrucous carcinoma
SOHND: Supraomohyoid neck dissection
VC: Verrucous carcinoma
